# Control of endemic swine flu persistence in farrow-to-finish pig farms: a stochastic metapopulation modeling assessment

**DOI:** 10.1186/s13567-017-0462-1

**Published:** 2017-10-03

**Authors:** Charlie Cador, Mathieu Andraud, Lander Willem, Nicolas Rose

**Affiliations:** 1 Swine Epidemiology and Welfare Research Unit, French Agency for Food, Environmental and Occupational Health & Safety (ANSES), BP 53, 22440 Ploufragan, France; 2 Centre for Health Economics & Modeling Infectious Diseases, Vaccine and Infectious Disease Institute, University of Antwerp Research, Antwerp, Belgium; 3 Université Bretagne Loire, Rennes, France

## Abstract

Swine influenza viruses (swIAVs) are known to persist endemically in farrow-to-finish pig farms, leading to repeated swine flu outbreaks in successive batches of pigs at a similar age (mostly around 8 weeks of age). This persistence in European swine herds involves swIAVs from European lineages including H1_av_N1, H1_hu_N2, H3N2, the 2009 H1N1 pandemic virus and their reassortants. The specific population dynamics of farrow-to-finish pig farms, the immune status of the animals at infection-time, the co-circulation of distinct subtypes leading to consecutive or concomitant infections have been evidenced as factors favouring swIAV persistence within herds. We developed a stochastic metapopulation model representing the co-circulation of two distinct swIAVs within a typical farrow-to-finish pig herd to evaluate the risk of reassortant viruses generation due to co-infection events. Control strategies related to herd management and/or vaccination schemes (batch-to-batch or mass vaccination of the sow herd and vaccination of growing pigs) were implemented to assess their relative efficacy regarding viral persistence. The overall probability of a co-infection event for France, possibly leading to reassortment, was evaluated to 16.8%. The export of consecutive piglets batches was identified as the most efficient measure facilitating swIAV infection fade-out. Although some vaccination schemes (batch-to-batch vaccination) had a beneficial effect in breeding sows by reducing the persistence of swIAVs within this subpopulation, none of vaccination strategies achieved swIAVs fade-out within the entire farrow-to-finish pig herd.

**Electronic supplementary material:**

The online version of this article (doi:10.1186/s13567-017-0462-1) contains supplementary material, which is available to authorized users.

## Introduction

Swine influenza A viruses (swIAVs) are polymorphic enveloped single-stranded RNA viruses from the *Orthomyxoviridae* family widespread in pig-production units throughout the world [1]. These viruses are of great economic importance for the swine industry because of their involvement as a major co-factor of porcine respiratory disease complex [2, 3] and being also responsible in some cases for severe pulmonary distress leading to growth retardations. In the past, mainly sporadic outbreaks, affecting a large part of the herd population in a relatively short time-interval but with short-term consequences at the herd scale, were reported. However, in recent years endemic forms of influenza infections have been increasingly documented with a global persistence of swIAV viruses at the herd scale, systematically affecting successive batches of growing pigs [4, 5]. As such, the burden of respiratory diseases in growing pigs due to bacterial co-infections is increasing and causes alarming use of antibiotics.

Three main subtypes have been circulating in swine populations worldwide: H1N1, H1N2 and H3N2 [6–8]. Those subtypes permanently evolve, ending in different lineages containing genetic components derived from both avian and human influenza A strains. To date, endemic swIAVs persistence in European swine herds includes H1_av_N1, H1_hu_N2, H3N2, the 2009 H1N1 pandemic virus and their reassortants. Weak cross-immunity between subtypes and the rapid spread of swIAVs within herds might cause multiple, and possibly concomitant, infections in one animal [4, 8, 9]. Co-infection events can lead to the emergence of reassortant viruses, potentially more pathogenic for animals and/or transmissible to humans, and are therefore recognized as a main threat for veterinary and public health [10–15].

Although the understanding of swIAVs transmission in pig populations is clearly pivotal to manage the risk of spillover to humans, Dorjee and collaborators highlighted the gap of knowledge on influenza dynamics at the pig farm-level [16]. Combining epidemiological, viral and immunological characteristics of swIAVs, mathematical models are comprehensive tools to analyze how the population dynamics and immunity influence the dynamics of influenza A virus infections [17–20]. To date, four modeling studies focusing on swIAV dynamics in swine production units have been published. Reynolds et al. [19] developed a deterministic model to assess the impact of vaccination strategies (e.g. mass and pre-farrowing vaccination) on the spread of influenza A infection in breeding and finishing herds based on parameter estimations from experimental conditions. Unless the vaccine and spreading strains were fully homologous, no vaccination strategy was able to eradicate the virus. This result is consistent with field observations where vaccination has only limited effect on the infection dynamics at the herd scale. Vaccination is commonly performed in breeding animals with two main objectives: the reduction of clinical expression in gestating sows and further delivery of maternally-derived antibodies (MDAs) for clinical protection of the offspring. Although the first objective is globally achieved, a recent study by Cador et al. [17] highlighted an ambiguous role of MDAs on the transmission dynamics. The presence of maternal immunity in young piglets was evidenced to extend the duration of the epidemics within batches, which in turn favored the transmission of the infection from batch to batch increasing therefore the persistence of swIAV at the population level. Pitzer et al. [18] used a stochastic model to analyze critical herd sizes for swIAV persistence according to herd type and management practices (variation of the between-birth interval and between two introductions of finishers) and showed that the swIAV was able to persist in relatively small populations. White et al. [20] recently proposed a stochastic model representing the infection dynamics of swIAV in a typical US farrow-to-wean production unit. The authors evaluated intervention strategies based on different vaccination schemes, biosecurity measures and management options and confirmed the role of piglets in swIAV persistence in breeding herds. These four modeling studies highlight the complex relationship between population dynamics, immunity and transmission dynamics of swIAV among a pig population, considering only one subtype.

Co-circulation of different swIAV subtypes within one swine herd is regularly observed in field conditions [4, 5] but has not yet been covered in modeling studies to date. Such phenomenon could increase the likelihood of virus persistence at the herd level triggering the risk of co-infection, possibly leading to reassortant viruses. In the present study we extended the stochastic event-based metapopulation model from Cador et al. [17] to represent co-circulation of two swIAVs in different farrow-to-finish pig farm settings in order to identify drivers responsible for viruses persistence at the herd level. The model was also used to assess the risk of co-infection events and to evaluate the impact of control strategies on the transmission dynamics and persistence of swIAVs at the herd scale based on (1) the implementation of different vaccination schemes and (2) the concurrent export of weaning piglet batches.

## Materials and methods

### Model structure

#### Population dynamics

Two subpopulations—breeding sows and growing pigs—are considered. Animals are subdivided into batches according to their physiological state (breeding sows) or age (growing pigs). The breeding sows iterate through three physiological states (service [32 days], gestation [82 days], lactation [26 days]), while growing pigs pass through three stages (lactation [21 days], nursery [51 days] and fattening [105 days]). Batches of animals are managed independently and housed in facilities according to their physiological or growing stage. Breeding sows’ facilities are divided in rooms containing batches having the same physiological stage. Growing pigs’ facilities are divided in rooms containing one single batch of animals with movements occurring at fixed times according to all-in-all-out management policy at the room level. Direct physical contacts between batches of sows and growing pigs only occur in the farrowing room, during the lactating stage. More information and a flow diagram of the farrow-to-finish pig farm can be found in Cador et al. [17]. The model was implemented in Matlab (MATLAB 2012b, TheMathWorks, Inc., Natick, Massachusetts, United States).

The original model considered one batch-rearing (denominated BR thereafter) management with seven batches (7-BR system) and was extended to account for several BR systems commonly used in the field [21]. Each BR system leads to specific population dynamics and herd structure with different numbers of batches and corresponding rooms in the different facilities of the herd (Table 1). Thus, in the BR systems with short between-batch intervals (10- and 20-BR systems; 2 or 1 week-interval, respectively), a high number of batches are housed in the same facility at the same time.

**Table 1:** **Modifications of the herd structure according to the BR (batch-rearing) systems** (from: Agriculture chamber of Brittany Region, 2017 [21])

	Values				
BR systems (number of batches)	4	5	7	10	20
Total number of productive sows	192	245	203	430	620
Proportion of herds according to the BR system	0.12	0.21	0.48	0.10	0.09
Duration of a sow reproductive cycle (days)	140	140	147^a^	140	140
Number of sows per batch	48	49	29	43	31
Number of piglets per batch	576	588	348	516	372
Interval between two successive batches of sows and pigs (days)	35	28	21	14	7
Number of batches in service room	1	2	2	3	5
Number of batches in gestating room	3	3	4	6	12
Number of farrowing rooms	1	1	2	2	4
Number of nursery rooms	2	2	2	4	7
Number of finishing rooms	3	4	6	9	18

^a^In the 7-BR system, weaning occurs at 28 days of age (21 days in the other BR systems). Then, the duration of a sow reproductive cycle is 7-day longer.

#### Epidemiological model

**Figure 1:**
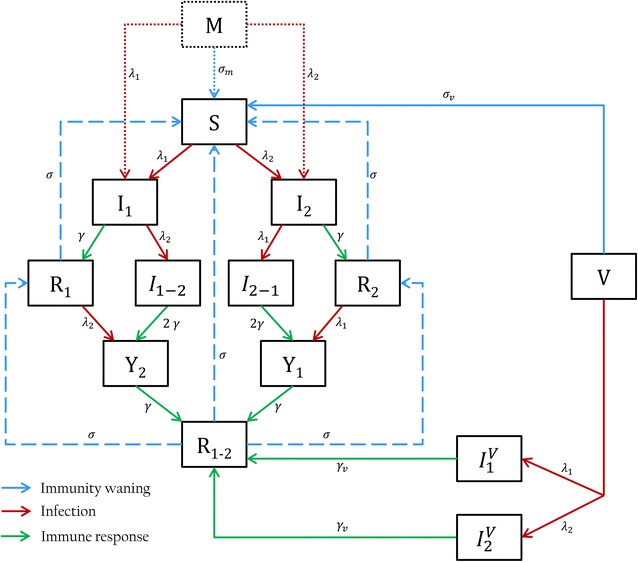
**swIAV infection states for breeding sows and growing pigs.** *S*: susceptible animals; *I*_1_ and *I*_2_: Infected animals by subtype 1 or subtype 2, respectively; *I*_1-2_ and *I*_2-1_: co-infected animals, *Y*_1_ and *Y*_2_: animals infected by subtype 1 or 2 while recovered from subtype 2 or 1, respectively; *R*_1_ and *R*_2_: animals recovered from subtype 1 or 2, respectively; *R*_1-2_: animals recovered to both subtypes; *M*: animals with maternally-derived antibodies; *V*: vaccinated animals; *I*_1_^*V*^ and *I*_2_^*V*^: animals infected by subtype 1 or 2 while vaccinated. Arrows represent the transitions between the classes. Dashed lines are specific to breeding sows. Dotted lines are specific to growing pigs. Solid lines are common to sows and growing pigs. Parameters involved in the transitions are summarized in Table 2.

Starting from a susceptible-infected-recovered-susceptible (SIRS) model, the epidemiological model in this study also accounts for specific features such as maternally-derived antibodies (MDAs) in neonates (lower susceptibility to infection) and sequential or concurrent infections with two different subtypes. The infectious process is ruled by the Gillespie’s direct algorithm [22], where each random event corresponds to a health transition of a single animal. Due to the fast-acting transmission between individuals, the batch was selected as epidemiological unit. No efficient cross-protection after sequential infections by two different subtypes has been evidenced neither in field nor experimental conditions [4, 8]. Therefore, no cross-protection has been implemented in the model allowing the animals to be co-infected by the two viral subtypes simultaneously or consecutively. swIAV infection states and health transitions are presented in Figure 1, denoting the swIAV subtypes by subscripts 1 and 2. Subsequent infections are explicitly included in the model to represent realistic durations of shedding periods and the development of strain-specific immunity. Animals can be infected by the second subtype while still shedding the first subtype (*I*_1-2_ or *I*_2-1_ classes) or after recovery from the first subtype given the absence of cross-immunity (*Y*_2_ or *Y*_1_ classes). Strain-specific immunity durations were assumed gamma-distributed with a mean duration of 180 days [17]. Sows infected by a second subtype (*Y*_2_ or *Y*_1_ classes) and recovered from the first one (*R*_1_ or *R*_2_ classes) are assumed to develop an immune response regarding the second strain (*R*_1-2_ or *R*_2-1_). The duration of dual immunity was restricted to 90 days after which immunity to the first subtype waned while specific immunity to the second strain persisted for 90 days (total immunity duration of 180 days). In consequence, breeding animals can be reinfected by the same subtype after waning immunity (Figure 1). Given the short lifespan of growing pigs (180 days), the loss of immunity towards a specific subtype with a subsequent re-infection by the same virus was deemed extremely unlikely. Hence, growing pigs were assumed to experiment only one infection for each subtype and develop specific immunity lasting for their economic lifespan. Waning of active and maternal immunity is represented by the stage approach [23–25]. The piglets’ immune status after colostrum intake is determined by the health states of the farrowing sows (denoted with superscript *i*). Recovered sows (*R*_1_^*i*^, *R*_2_^*i*^, *R*_1-2_^*i*^, *R*_2-1_^*i*^ and *R* ^*i*^) give birth to *M* ^*i*^ piglets; *Y*_1_ and *Y*_2_ sows to *M* ^*i*=1^ piglets and *S*, *I*_1_, *I*_2_, *I*_1-2_ and *I*_2-1_ sows to fully susceptible piglets *S*. Piglets with MDAs (*M* ^*i*^ classes) were assumed to be partially protected against infection through a reduced susceptibility [26]. This protective factor was considered strain-specific based on the proportion of farrowing sows immune to each subtype.

Merging this epidemiological model with the population dynamics resulted in an event-driven epidemiological stochastic model embedded in specific population dynamics.

### Implementation of vaccination in the swIAV infectious model

The trivalent vaccine used in European countries covers the majority of the circulating strains in Europe [27]. In our model, vaccination provides partial protection against the two circulating subtypes and is ineffective when applied to actively shedding animals (*I* and *Y* classes) because the immunity onset is established after 7 days at least after primary vaccination (RESPIPORC FLU3, summary of product characteristics, [28]). Vaccinated susceptible and immune animals (*S* and *R* classes, respectively) develop vaccine-induced immunity against both viral subtypes and are represented by *V* classes. Waning of vaccine-induced immunity was assumed gamma-distributed and modeled using 7-exponential classes (stage approach). Vaccinated pigs have a reduced swIAV transmission probability and a lower shedding period [29]. In addition, piglets with high antibody levels (*M* ^*i*=1^ to *M* ^*i*=3^ stages) were assumed not to react to vaccination due to interference between passive- and vaccine-induced immunity in young piglets [30]. Conversely, the vaccination of piglets with lower antibody levels (*M* ^*i*=4^ to *M* ^*i*=7^ stages) induced an increased duration of the vaccine-induced immunity according to the antibody decay of the piglets. Vaccinated animals are assumed to experience at most one infection by either subtype after which a long-lasting immunity to both subtypes is established until slaughter age [31].

### Force of infections

As swIAV transmission occurs mainly through pig-to-pig contact and the exposure to contaminated aerosols, the force of infection applied to each animal includes (1) the direct transmission from infected animals within the room and (2) the indirect transmission through airborne route from infected animals within the entire facility. Subtype-specific forces of infection are calculated combining direct and indirect transmission routes. Parameter definitions and values are provided in Table 2.

**Table 2:** Parameters used in the swIAV infection dynamics model (Figure 1)

Rate	Event	Sources	Value
β	Direct transmission rate	Cador et al. [26]	2.43
γ	Recovery rate for infected animals (days^−1^)	Rose et al. [4]	1/8.5
*β*_*air*_	Between-batch transmission rate	Cador et al. [17]	0.1
*ɛ*	Susceptibility to infection for piglets having MDAs	Cador et al. [26]	0.39
*σ*	Immunity waning (days^−1^)	Cador et al. [17]	1/180
*σ*_*m*_	Loss of maternal immunity (days^−1^)	Cador et al. [26]	1/70
*β*_*v*_	Transmission rate due to vaccine-immune infected animals	Romagosa et al. [29]	0.28
*γ*_*v*_	Recovery rate for infected-vaccinated animals (days^−1^)	Romagosa et al. [29]	1/4.0
*σ*_*v*_	Vaccine immunity waning (days^−1^)	Projected from Cador et al. [26]	1/105

The within-room direct transmission force of infection *λ*_*i*_^*direct*^(*t*, *r*) for subtype *i*, at time *t* in room *r* is defined as:

$$\lambda_{i}^{direct} \left( {t,r} \right) = \frac{{\beta (I_{i} \left( {t,r} \right) + I_{ji} \left( {t,r} \right) + I_{ij} \left( {t,r} \right) + Y_{i} \left( {t,r} \right)) + \beta_{v} I_{i}^{v} \left( {t,r} \right)}}{{N_{r}^{{}} }},\quad j \ne i.$$

*I*_*i*_ represents the number of animals infected by subtype *i* only, *I*_*ij*_ and *I*_*ji*_ the number of animals infected by both subtypes simultaneously (accounting for the sequence of infections) and *Y*_*i*_ the number of animals infected by subtype *i* while immune against *j*. *I*_*i*_^*v*^ corresponds to the number of animals infected by subtype *i* while vaccinated. *β* and *β*_*v*_ denote the related transmission rates per day for non-vaccinated and vaccinated pigs, respectively. Transmission rates for both subtypes are assumed to be the same.

The between-room airborne force of infection *λ*_*i*_^*indirect*^ (*t*, *r*) for subtype *i*, at time *t* in room *r* is expressed from the total prevalence of infected animals by subtype *i* at time *t* in neighbouring rooms $r^{\prime}$:

$$\lambda_{i}^{indirect} \left( {t,r} \right) = \beta_{air} \frac{{\mathop \sum \nolimits_{r' \ne r}^{{}} \left( {I_{i} \left( {t,r'} \right) + I_{ji} \left( {t,r'} \right) + I_{ij} \left( {t,r'} \right) + Y_{i} \left( {t,r'} \right) + I_{i}^{v} \left( {t,r'} \right)} \right)}}{{\mathop \sum \nolimits_{{r^{'} \ne r}}^{{}} N_{r'} }}.$$

Here, *β*_*air*_ denotes the transmission rate by airborne route and *N*_*r*’_ the total number of pigs in the other rooms $r^{\prime}$ of the same facility. This airborne force of infection is also applied to susceptible animals during transfer from one facility to another [17].

Therefore, the global force of infection *λ* (*t*, *r*) for a given subtype *i* at time *t* in room *r* is:

$$\lambda_{i} \left( {t,r} \right) = \lambda_{i}^{direct} \left( {t,r} \right) + \lambda_{i}^{indirect} \left( {t,r} \right) .$$

The force of infection for animals with MDAs is assumed to be $\varepsilon *\lambda_{i} \left( {t,r} \right)$ using a reduced susceptibility factor *ɛ* [26].

### Initialisation and study design

Subtypes were introduced separately with a lag-time of 20 weeks. The first subtype was introduced in a fully susceptible and demographically stable herd by the importation of a shedding gilt during the replacement process in the first batch between the farrowing and service room. Assuming no cross-immunity between the two subtypes, the population remained fully susceptible to the second subtype. Introduction of the second subtype was performed as described for the first subtype. Simulations were run for 5 years after the second virus introduction. For each scenario, 200 simulations are performed to capture the variability induced by stochastic processes while keeping a reasonable simulation time.

### Impact of the BR system on swIAV persistence

The impact of the different BR systems has been evaluated regarding the time to swIAVs fade-out and the probability of co-infection events. The latter risk was approximated as the proportion of days with co-infections on the total number of days with infected animals for each BR system (daily probability of co-infection). Finally, the global probability of co-infection events was assessed by combining the proportion of simulations with co-infections with the daily probability of co-infection occurrence, weighted by the relative proportion of each BR system in the population in France [21].

### Implementation of control strategies

We tested 13 different combinations of vaccination schemes and piglet batch export. Each scenario has been simulated for two extreme BR systems in terms of batch population-size and time interval between batches (5- and 20-BR).

#### Vaccination

Vaccination is implemented 3 months after the introduction of the second subtype.

In the present study, three vaccination schemes were considered:

- *Batch-to-batch vaccination of the breeding sows.* Vaccination is implemented in the gestating room 15 days before farrowing on all animals from the batch expecting to farrow. Hence vaccination time is ruled by the physiological status of the sows and the different batches are desynchronized in terms of boost vaccine immunity. This vaccine strategy aims at inducing a high antibody level in colostrum (pre-farrowing vaccination) and further transfer to piglets.
- *Mass vaccination of the breeding sows.* Vaccination is implemented every 3 or 4 months for all breeding sows present in service, gestating and farrowing rooms at the same time, in order to reduce infection pressure in breeding sow facilities.
- *Batch-to-batch vaccination of the breeding sows and growing pigs.* In addition to batch-to batch vaccination of the sows, growing pig vaccination is implemented in the five first batches entering the nursery from the beginning of the vaccination program to reduce the infection pressure in growing pig facilities. The same scenario was also tested on the five first batches entering the finishing rooms.

#### Export of batches of weaned piglets

Batch export is implemented 3 months after the introduction of the second subtype. We tested the export of one batch of weaned piglets at a regular interval (every 24 weeks). The time interval between 2 exports was chosen to represent the export of a whole batch of weaned piglets to an external wean-to-finish site reared on an all-in all-out principle. The export of consecutive batches (2 or 4 batches according to the BR system) has also been tested.

#### Statistical analysis of scenarios outputs

The efficiency of the different control strategies was evaluated as regards the probability of swIAVs fade-out within the herd. Time to swIAVs fade-out was studied using survival analysis comparing survival curves corresponding to different strategies using log-rank test. When conditions of proportional hazards assumption were met, a Cox-proportional hazard model was used to estimate Hazard ratios (HR) and compare control scenarios to the baseline (no measure implemented).

## Results

### Description of simulations after introduction of the two subtypes

**Figure 2:**
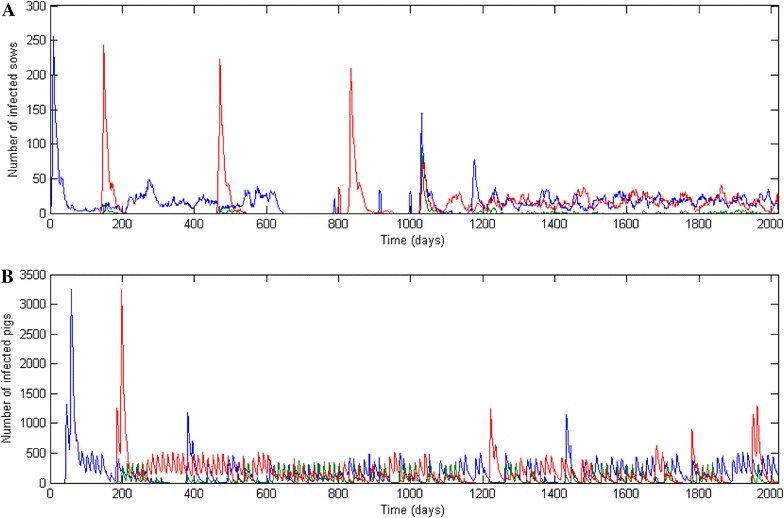
**swIAV spread within a farrow-to-finish herd (10-BR system) after introduction of the two subtypes (140 days apart).** Example of one simulation in the sow herd (**A**) and the growing pig part (**B**). The blue line represents the animals infected by subtype 1, the red line the animals infected by subtype 2 and the green line the co-infected animals.

Virus introduction via an infectious gilt (on D0 and D140) caused an initial peak in the number of infected sows due to the fully susceptible population (Figure 2). Transmission events to growing pigs occurred in the farrowing site, triggering the virus spread into the nursery and the finishing facilities. After an initial large outbreak in growing pigs and breeding animals, virus persistence was observed at the herd level due to the constant introduction of susceptible animals and immunity decay. However, sporadic fade-out periods were alternatively observed in growing pig and breeding sow subpopulations. These periods remained of relatively short durations due to virus transfer from one subpopulation to the other during between-facility movements (example at D780 in Figure 2).

**Figure 3:**
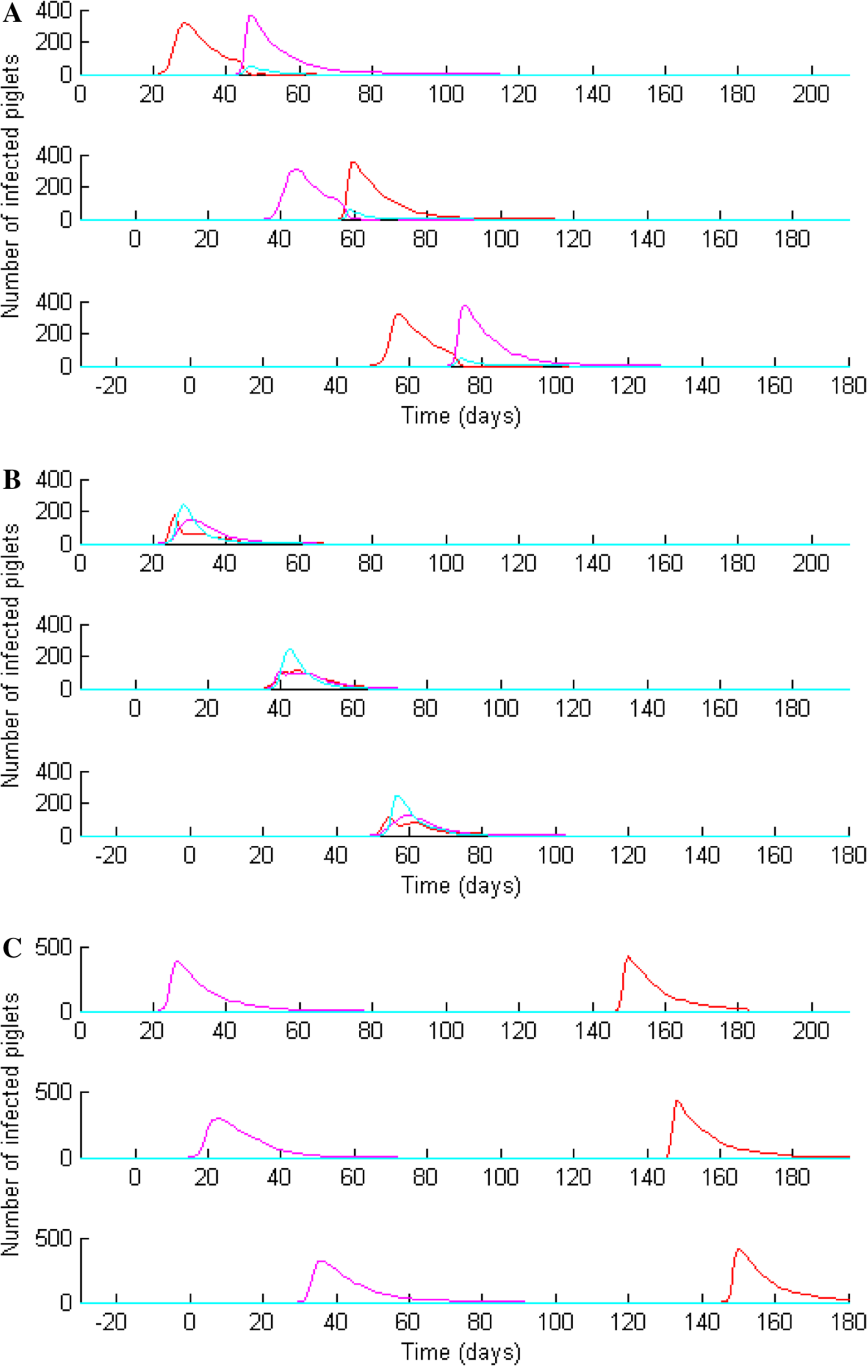
**Evidence of different patterns of swIAV co-circulation within growing pigs leading to partial and total concomitant infections (A, B), or consecutive infections (C).** Example of simulations carried out in the 10-BR system.

In growing pigs and at the batch level, swIAVs infections of piglets occurred on successive batches at a similar age (i.e. around weaning). Consecutive batches showed similar outbreaks (Figure 3, three consecutive batches reared in a 10-BR system) however different patterns could be observed regarding the co-circulation of subtypes: (a) the infection by the subtype *i* closely followed by the infection by subtype *j* (or vice versa), allowing co-infections of the piglets during a short overlapping time-interval (Figure 3A); (b) the strict concomitant infections by the subtypes *i* and *j*, inducing a moderate number of infections by each subtype separately but a great number of co-infected piglets (Figure 3B); (c) the infection by the subtype *i* in young piglets followed by the infection of subtype *j* a few months later in finishing rooms, inducing two distinct outbreaks (Figure 3C). In each scenario, between 60 and 75% of the piglets were infected at the epidemic peak.

### Impact of the BR system on swIAVs dynamics

#### Impact of the BR system on the global within-herd persistence

**Figure 4:**
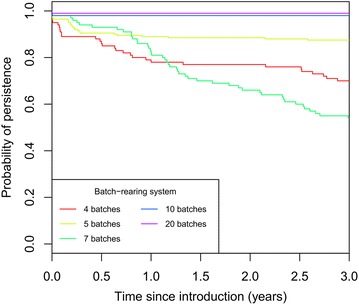
**Survival analysis of swIAV within-herd fade-out according to the batch-rearing system (4-, 5-, 7-, 10- or 20-BR system).** 200 simulations per scenario, χ2 Log rank test = 130, 4 df, *p* < 0.001.

Survival analysis of swIAV persistence at the herd level showed a low probability of infection fade-out up to 5 years after introduction (Figure 4). In the absence of external reintroduction and for all BR systems, at least one swIAV subtype was found to persist for more than 3 years with a probability of 60% whatever the BR system. Differences between BR systems were however significant (*p* < 0.001, log-rank test). For BR systems with short intervals between batches (10 and 20 batches with a 14- and 7-days interval respectively), we observed a systematic endemic persistence. For these BR systems, only 8 out of 400 simulations showed stochastic fade-out before virus transmission while the infectious process lasted up to 5 years (simulation time) in all the other simulations (Table 3). Coinfections in growing pigs occurred in 84% of the 200 simulations in herds managed according to 10 and 20 batch-rearing systems.

**Table 3:** **Summary statistics (percentage of simulations) of the circulation pattern in growing pigs (no virus spread after introduction, spread of each subtype alone, spread of both subtypes with co-infection events, 5 batch-rearing (BR) systems, 200 simulations per BR system)**

	No virus spread	First subtype only (%)	Second subtype only (%)	Both subtypes (with co-infection events) (%)
4-BR system	11	23	19	47 (45)
5-BR system	10	21.5	22.5	46 (46)
7-BR system	5.5	18	24	52.5 (49)
10-BR system	2	5	9	84 (84)
20-BR system	1	7	8	84 (84)

The two BR systems with the largest between-batch intervals (4- and 5-BR with a 35- and 28-days interval respectively) showed similar behavior with a 10% fade-out probability in the first months after introduction followed by a slow decay of persistence probability throughout the simulation-time. While displaying a higher fade-out probability than the other BR systems in the two first months after introduction due to the longest in-between batches intervals, the average probability of persistence after 5 years was evaluated to 61 and 86% for herd managed according to 4- and 5-BR systems, respectively. Forty-five to 46% of simulations resulted in coinfection events in growing pigs, reducing the probability of coinfection by 1.8 when compared to intensive batch-rearing systems (10-BR and 20-BR). The co-circulation of both subtypes was nevertheless still more frequent than the circulation of a unique subtype in these BR systems. The 7-BR system (21-day interval) showed an intermediate behavior regarding the coinfections events at the herd level, occurring in 49% of simulations (Table 3). Although swIAVs were more likely to persist during the first year after introduction compared to the 4- and 5-BR systems, the 7-BR system showed a continuous decay over time reaching the lowest probability of swIAV persistence after 5 years post-introduction (35% on average; data not shown). The order of subtype introduction did not have any effect with similar proportions of the simulations showing the circulation of only the first or the second subtype, irrespectively of the BR system (Table 3).

#### Assessment of the frequency of co-infection events according to the BR system

**Figure 5:**
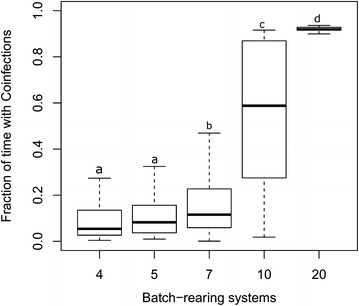
**Boxplots representing the occurrence of co-infections in growing pigs (number of days with co-infected pigs/number of days with infected pigs) according to the batch-rearing system (4-, 5-, 7-, 10- or 20-BR system) (Kruskal–Wallis test,** ***p*** **<** **0.001).** Different letters indicate significant differences between BR systems (Wilcoxon rank sum test for pairwise comparisons).

Co-circulation of both subtypes can lead to co-infections (green lines, Figure 2). The BR system had a significant impact on the presence of co-circulations at the herd level and co-infection at the individual level (Table 3). Likewise, the probability of co-infection events was significantly different between the BR systems (Kruskal–Wallis test, *p* < 0.001) (Figure 5). As such, the median occurrence of co-infections was 5.4, 8.1 and 16.4% for the 4-, 5- and 7-BR systems, respectively, compared to 58.8 and 91.9% in the 10- and 20-BR systems. The 10-BR system presented the highest dispersion in the occurrence of co-infections. When accounting for the relative proportion of each BR system in the French pig herd populations, the overall probability of a co-infection event possibly leading to reassortment was 16.8%.

### Evaluation of control measures

#### Impact of vaccination on swIAVs persistence in breeding sows

The implementation of vaccination 3 months after the introduction of the second subtype induced a significant rise of fade-out probability in breeding sows in the 5-BR system while having no impact on the persistence in breeding sows reared in 20-BR systems (see Additional file 1).

In the 5-BR system, all vaccination schemes significantly increased swIAV fade-out probability (HR = 4.3 [3.1–5.9] for batch-to-batch vaccination, HR = 1.6 [1.2–2.1] for mass vaccination, Cox proportional hazard model, *p* values < 0.05). No significant difference was observed in regards with mass vaccination schedule (3 or 4 month-interval) and outputs from these two vaccination schemes have been merged for further analyses. Batch-to-batch vaccination led to a rapid decrease of swIAV persistence probability a few months after implementation while the mass vaccination had a limited impact at that time. Mass vaccination reduced swIAV persistence probability at regular intervals but was still less efficient in sows 4 years after introduction compared to the batch-to-batch vaccination.

#### Impact of vaccination on swIAV persistence at the herd level

**Figure 6:**
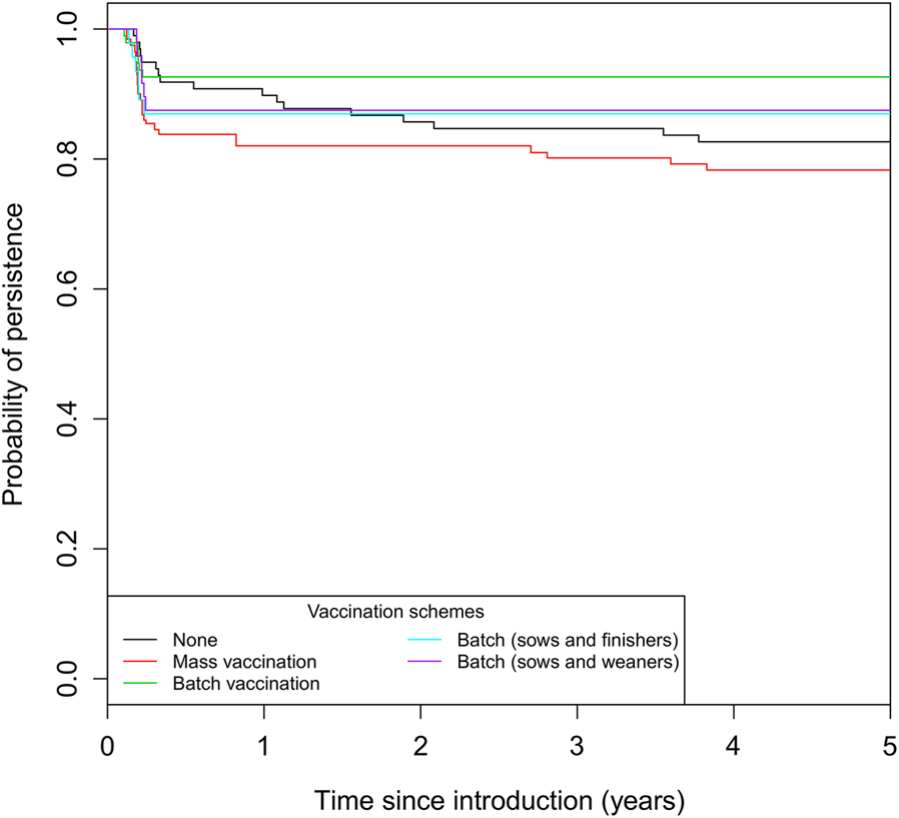
**Survival analysis of swIAV within-herd fade-out in pig herds reared in 5-BR system according to the vaccination scheme.** Batch-to-batch vaccination in sows, sows and five consecutive batches of weaned piglets, sows and five consecutive batches of finishing pigs, and mass vaccination. 200 simulations per scenario. χ2 Log rank test = 13.8, 4 df, *p* < 0.05.

Although vaccination increased swIAV fade-out probability in breeding sows, the effect on the global persistence of swine flu was not reflected at the herd level. In the 5-BR system, although a global difference was found between the tested scenarios (Figure 6), when each vaccination strategies was compared to the reference ‘No control measures’, no significant differences were found (Cox proportional hazard model, *p* > 0.05). Vaccination strategies had no impact on global swIAV persistence in the 20-BR system (data not shown).

#### Impact of the association of vaccination and batch export on swIAV persistence at the herd level

**Figure 7:**
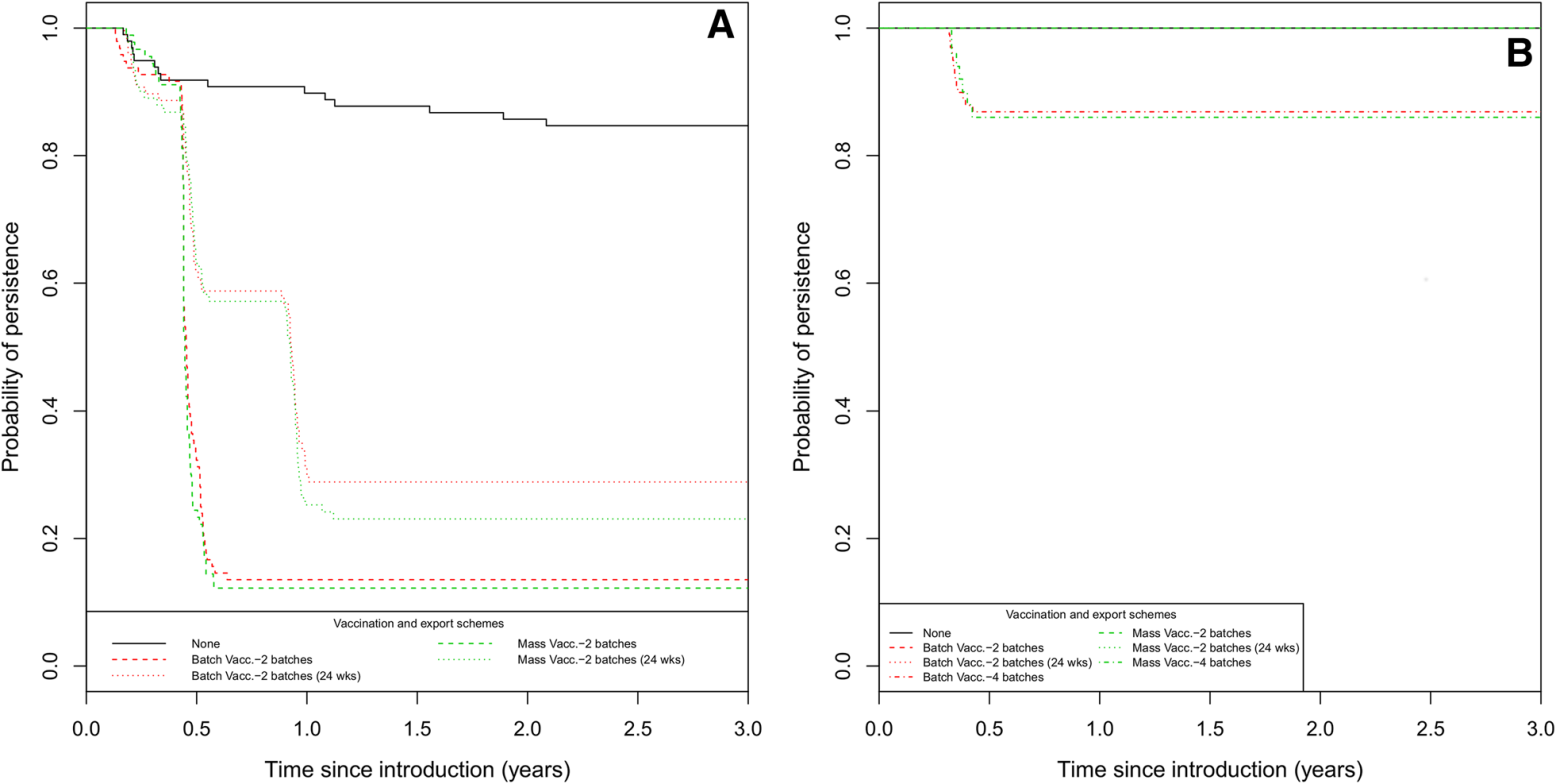
**Survival analysis of swIAV within-herd fade-out in pig herds reared in 5-** (**A**) **and 20-BR system** (**B**) **according to the export of piglets batches (consecutively [2 or 4 batches], or at a fixed-interval [24** **weeks apart] representing the export of a single batch to a wean-to-finish site).** 200 simulations per scenario. χ2 Log rank test = 229, 6 df, *p* < 0.001.

In the 5-BR system, the export of piglet batches in vaccinated herds increased the probability of swIAV fade-out compared to vaccination alone (Figure 7A). The concurrent export of two successive batches led to a higher probability of fade-out than the export of two batches 24 weeks apart, e.g. within the batch-to-batch vaccination scheme: HR = 13.0 [7.6–22.0] vs. HR = 6.2 [3.65–10.5], respectively, taking the “No measure” scenario as the baseline (Cox proportional hazard model, *p* values < 0.05). No significant difference was observed between the vaccination schemes when combined with export of weaned piglets (Figure 7A). In the 20-BR system, the simultaneous export of four consecutive batches was required to achieve a probability of swIAV within-herd fade-out of 14% (Figure 7B).

## Discussion

In this study, we extended a validated modeling framework to investigate swIAV transmission dynamics of two subtypes within a farrow-to-finish pig herd and to evaluate control strategies to prevent endemic persistence. Our model simulations showed an almost-mechanic repetition of swIAV outbreaks with infections in successive batches of piglets at a similar age. This pattern has also been shown in field conditions [4] by studying three endemically infected herds. Co-circulation of two distinct subtypes (H1_av_N1 and H1_hu_N2) was observed at the batch level and/or at the individual level, resulting in two distinct outbreaks or episodes with co-infections and reassortant viruses. They also identified a faster spread of the virus in pigs in finishing rooms compared to nursery rooms. Simulations from our model are consistent with this behaviour with a sharper epidemic peak in the three outbreaks occurring in older animals although the transmission characteristics between both subtypes were the same in our model. When two influenza outbreaks affect animals consecutively (slight overlap), the order of subtype infection changed from batch to batch. This phenomenon has not been shown in field conditions to date but could contribute to the understanding of repeated infections from batch to batch.

The present model highlighted the difficulty in containing transmission once a virus is introduced within the farm, consistently with White et al. [20]. Indeed, independently from the batch-rearing system, at least one of the two introduced viruses was still circulating 5 years after a unique introduction in 78% of the simulations. However, some differences were observed according to the BR systems. The 10- and 20-BR systems, characterized by short between-batch intervals and a large herd size (430 and 620 sows, respectively), inducing a huge number of animals in nursery and fattening facilities, showed a systematic persistence of swIAVs after introduction. However, both rearing systems displayed different occurrence of co-infections. Although both subtypes were circulating in the majority of the cases, a higher variability was associated with the 10-BR system due to an important number of simulations with a long persistence of a unique subtype. Moreover, the occurrence of co-infection events appeared sporadic as compared to 20-BR system when both subtypes were circulating within the herd (median duration of the presence of both subtypes: 767 vs. 1831 days in the 20-BR system) with temporary switch between subtypes. However, because of the size of the population and the frequent introduction of susceptible animals (every 2 weeks) in this BR system, the persistence of a unique subtype for 5 years occurred more frequently than in BR systems with larger between-batch intervals.

The batch-rearing systems with the largest between-batch intervals (4 and 5 batches) have intrinsic specific characteristics that favour swIAV fade-out on top of the interval duration between batches. Fade-out of the virus in 10% of the simulations in the month following the introduction was due to the particular structure of the pig herds reared with the 4-BR system. Indeed, there is a unique batch in service room in herds reared with this system. Therefore, as sows are housed in this facility during 33 days and the virus was introduced at the entrance of the animals within the room, the epidemic outbreak could resume before the end of this period. Thus, recovered sows entering in gestating rooms couldn’t initiate the infectious process in other batches. The fade-out of the virus in around 10% of the simulations 1 month later in pig herds reared in the 5-BR system was also likely due to the structure of the herd. Although two batches are housed in the service room at the same time helping the transmission of the virus in the gestation room, the duration until the first entrance of pregnant sows in farrowing room is 23 days (only 9 days in the 4-BR system), yielding to the possible termination of the infectious process in the gestation room with a less likely spread to the farrowing rooms.

Once the virus introduced in growing pigs, a higher persistence was observed in the 4- and 5-BR compared to the 7-BR system probably due to the batch size (48 and 49 vs. 29 sows per batch respectively) and a different weaning age (21 vs. 28 days-old), which could favour fade-out in farrowing rooms before nursery entrance. The persistence could also be enhanced in the 5-BR system by the larger number of animals in the herd (245 total sows vs. 192 and 203 in the 4- and 7-BR systems). A modeling study focusing on the circulation of one influenza strain corroborates this effect of population size on swIAV persistence [18]. The choice was made to represent each BR system with the corresponding average herd size from statistics available at the country level [21]. Another option could have been to control for herd size and to evaluate only the impact of the herd organization and the time-interval between batches. However, this would have led to unrealistic situations as the choice of the BR system is governed partly by the herd size (large herds are generally reared using a 10- or 20-BR system). Moreover, representing realistic situations showed that the time-interval between batches is not the only determinant of swIAV persistence but the batch size and possibly the weaning-age also have an impact on infection dynamics.

The development of a model representing co-circulation of two swine influenza strains within pig herds had not been carried out before while models on the co-circulation of influenza A virus strains in humans exist [32, 33]. The model developed by Moghadas et al. [32] allowed consecutive infections of individuals assuming a partial cross-immunity after a first infection. In our model, no cross-immunity between subtypes was included as all the available experimental [34, 35] or field data [4] suggested an extremely limited cross-reactivity between the main subtypes known to endemically co-infect swine herds in the EU context (H1_av_N1 and H1_hu_N2). Zhang et al. [33] modelled the possibility of co-infections and the generation of a reassortant strain at a rate function of the days with co-infections. Only co-infection events were represented in our model as data were lacking to parameterize the likelihood of reassortant generation in case of co-infection in the swine context. Experimental in vitro, ex vivo and in vivo studies would be required to further parameterize this phenomenon. However, using our co-infections event as a proxy and combining the proportion of simulations with co-infections with the daily probability of occurrence, weighted by the relative proportion of each BR system in the population, the probability of co-infection events (16.8%) was found higher than the actual proportion of reassortant strains reported by the national surveillance system of swine influenza infections in French herds (3–5% according to years; [36]). The difference might be due to the probability of successful virus reassortment, which is not accounted in our analysis.

Control strategies were evaluated based on two BR systems selected among the five tested. According to the global spread of the virus, two subsets of BR systems were distinguished with similar behaviors obtained with the 4-, 7- and 5-BR systems, and with the 10- and 20-BR systems, respectively. To assess the impact of control measure, the 5- and 20-BR systems resulting in the longest swIAV persistence has been selected within each subset.

A key finding of this study was the selection of the export of piglet batches as the most effective measure favoring infection fade-out. The number of batches to export to obtain a significant effect depended on the BR system. Indeed, the export of four consecutive batches was required in the 20-BR system while the export of a single batch in the 5-BR system was sufficient to observe a significant decrease of swIAV persistence. The theoretical export of four consecutive batches appears difficult to set up in field conditions but highlights the necessity to insert “gaps” in the growing pigs facilities in order to block the swIAV infection dynamics. In this type of large herds with short between-batch intervals, a solution could be to breed the minimum of batches on the main breeding site and to export, if possible, other batches to another wean-to-finish site. Indeed, lower swIAV persistence has been observed in specialized growing sites compared to farrow-to-finish pig herds [18, 37]. The consecutive export of piglet batches also led to an increased virus extinction compared to a regular export (24 weeks apart), suggesting a synergic effect on the infection disruption of consecutive exports. This is probably due to the longer period without introduction of a new cohort of potentially susceptible piglets in nursery.

Our model indicates that none of current vaccination strategies were sufficient to eliminate influenza in farrow-to-finish pig herds although it is a frequent measure implemented for swIAV control in pig herds [38]. Similarly to other modeling studies [19, 20], mass vaccination as well as batch-to-batch vaccination alone did not significantly reduce swIAV within-herd persistence. Although vaccination was found to favour swIAV fade-out in breeding sows in large between-batch interval BR systems, it had no impact on the global swIAV persistence. Indeed, even when sows were vaccinated, the viruses could still circulate and be maintained in the growing pig subpopulation and be further reintroduced in the breeding part of the herd when sows and piglets have contact (e.g. weaning stage). Usually carried out at the end of gestation, the aim of the batch-to-batch vaccination is to protect sows against reproductive disorders and to deliver maternal immunity to neonatal piglets. However, the adverse effect of maternal immunity previously highlighted [17] suggested thinking flu vaccination in swine operations differently. Incidentally, the batch-to-batch vaccination of sows, although allowing a 10% infection fade-out probability after implementation compared to the control scenario, did not affect the probability of fade-out afterwards, probably because the impact was counterbalanced by the adverse effect of maternal immunity in piglets. The mass vaccination was expected to confer a herd immunity to the sows [39] but the model showed a better efficiency of the batch-to-batch vaccination in breeding sows. This might be due to the systematic boost of immunity in sows a couple of weeks before farrowing preventing the relaunch of the infectious process in the breeding herd by growing pigs. White et al. [20] showed that early weaning of piglets after 0–7 days of age reduced endemic prevalence in farrow-to-wean units. Because such early-weaning is not allowed in Europe (Council Directive 91/630/EEC), we evaluated the export of 3-week-old piglets aiming at breaking the infectious process in growing pig population in combination with vaccination schemes. When the export of batches was implemented, the mass vaccination appeared more helpful to decrease swIAV within-herd persistence. Conversely, no differences between vaccination schemes were observed in the case of the concurrent export of consecutive batches, probably because of the limited effect of vaccination within the large impact of the export. The additional vaccination of five consecutive batches (deemed as the maximum affordable) of growing pigs or finishers did not significantly increase swIAV fade-out and could not mimic the effect of growing-pigs batches exports.

In the present model, the vaccination of the animals induced a tenfold lower transmission rate and a twofold reduction of the duration of the shedding period compared to fully-susceptible animals [29]. To the best of our knowledge, no quantitative data on the amount of virus shed by vaccinated animals have been published to date. Thus, this reduction of transmission could be due to a reduction of susceptibility in piglets having vaccine-induced immunity. A tenfold lower susceptibility has been tested with the present model but model outcomes as regards vaccine impact were not modified (data not shown). However, parameterization of the vaccine effect was made using the only data available and corresponding to a US vaccine evaluated in front of a US strain challenge. Further data should be collected on vaccine efficacy regarding the reduction in transmission and/or susceptibility to infection in the EU context to consolidate our conclusions.

swIAV endemic persistence in farrow-to-finish herds was shown in this study to be determined by multiple characteristics, which are not independent. Hence the choice of a BR system involves a specific herd structure, subpopulation sizes and between-batch time intervals. They all participate to a different degree to the persistence of the infectious process. As such, the advantage of long intervals between batches is possibly counterbalanced by large subpopulations. The observed chronic persistence of swIAVs at the herd level in field conditions and the difficulty to eradicate the infection once introduced even using different vaccination programs can be understood in the light of the present study. Control and progressive eradication of the infection requires combined vaccination programs adapted to the BR system in association with rearing practices aiming at introducing gaps in the growing part of the herd and an adequate separation between the breeding and the growing part of the herd to prevent reactivation.

## Additional file

**Additional file 1.** **Survival analysis of swIAV fade-out in breeding sows reared in the 5- (A) or 20-BR system (B) according to the vaccination scheme (batch-to-batch or mass vaccination every 3 or 4 months)**. 200 simulations per scenario, χ2 Log rank test = 121, 3 df, *p* < 0.001.
