# Supplementary figures and images for: Control of endemic swine flu persistence in farrow-to-finish pig farms: a stochastic metapopulation modeling assessment
Source: Vet Res. 2017 Oct 3;48:58. doi: 10.1186/s13567-017-0462-1 (PMC5627436; doi:10.1186/s13567-017-0462-1)

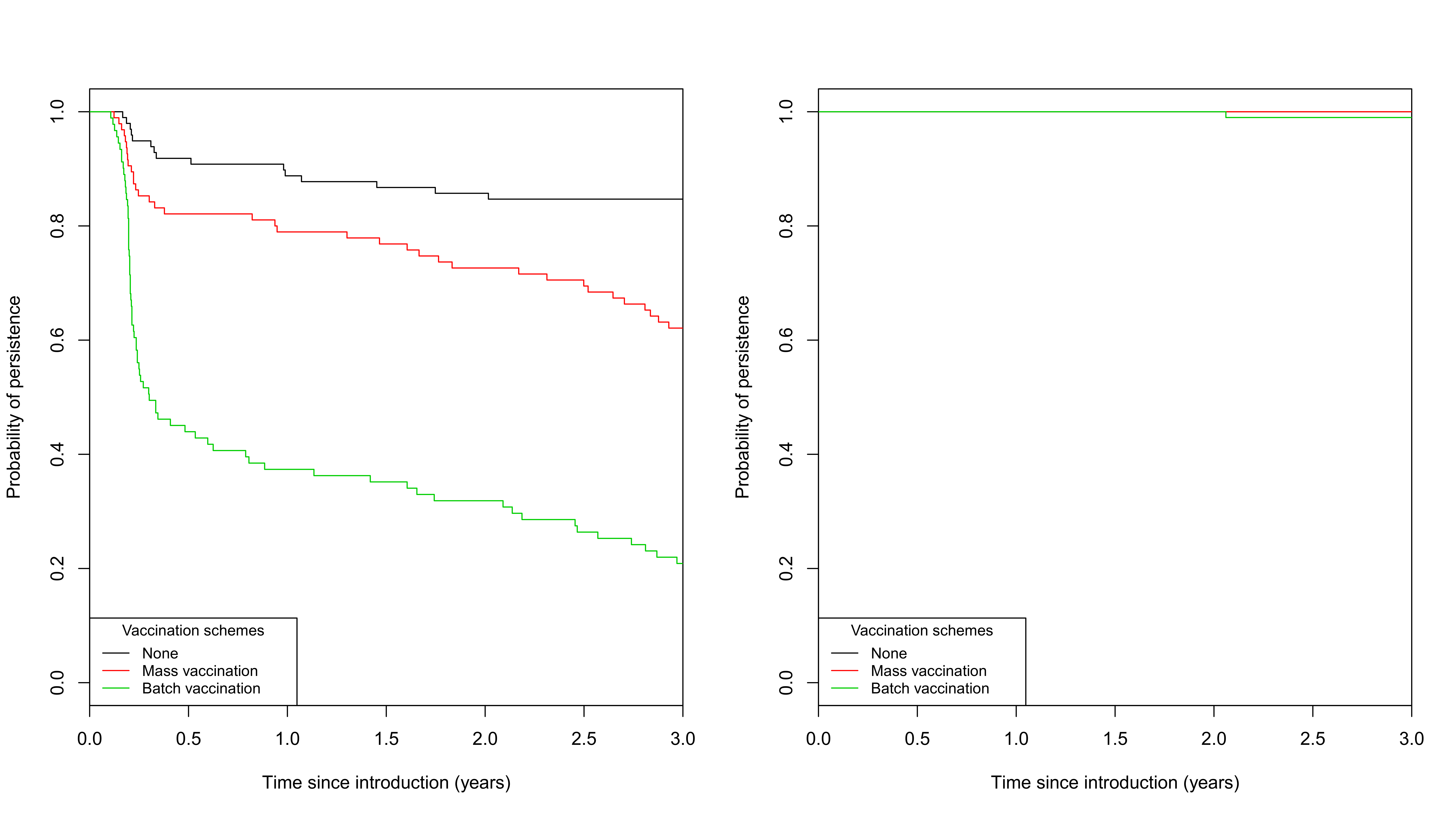

Supplement: Supplementary file 1 — Additional file 1. Survival analysis of swIAV fade-out in breeding sows reared in the 5- (A) or 20-BR system (B) according to the vaccination scheme (batch-to-batch or mass vaccination every 3 or 4 months). 200 simulations per scenario, χ2 Log rank test = 121, 3 df, p < 0.001. [file 13567_2017_462_MOESM1_ESM.tiff]
